# Dietary garlic and hip osteoarthritis: evidence of a protective effect and putative mechanism of action

**DOI:** 10.1186/1471-2474-11-280

**Published:** 2010-12-08

**Authors:** Frances MK Williams, Jane Skinner, Tim D Spector, Aedin Cassidy, Ian M Clark, Rose M Davidson, Alex J MacGregor

**Affiliations:** 1 Dept Twin Research and Genetic Epidemiology, King's College London, London, UK; 2 School of Medicine, Health Policy and Practice, University of East Anglia, Norwich, UK; 3 School of Biological Sciences, University of East Anglia, Norwich, UK

## Abstract

**Background:**

Patterns of food intake and prevalent osteoarthritis of the hand, hip, and knee were studied using the twin design to limit the effect of confounding factors. Compounds found in associated food groups were further studied *in vitro*.

**Methods:**

Cross-sectional study conducted in a large population-based volunteer cohort of twins. Food intake was evaluated using the Food Frequency Questionnaire; OA was determined using plain radiographs. Analyses were adjusted for age, BMI and physical activity. Subsequent *in vitro *studies examined the effects of allium-derived compounds on the expression of matrix-degrading proteases in SW1353 chondrosarcoma cells.

**Results:**

Data were available, depending on phenotype, for 654-1082 of 1086 female twins (median age 58.9 years; range 46-77). Trends in dietary analysis revealed a specific pattern of dietary intake, that high in fruit and vegetables, showed an inverse association with hip OA (p = 0.022). Consumption of 'non-citrus fruit' (p = 0.015) and 'alliums' (p = 0.029) had the strongest protective effect. Alliums contain diallyl disulphide which was shown to abrogate cytokine-induced matrix metalloproteinase expression.

**Conclusions:**

Studies of diet are notorious for their confounding by lifestyle effects. While taking account of BMI, the data show an independent effect of a diet high in fruit and vegetables, suggesting it to be protective against radiographic hip OA. Furthermore, diallyl disulphide, a compound found in garlic and other alliums, represses the expression of matrix-degrading proteases in chondrocyte-like cells, providing a potential mechanism of action.

## Background

Osteoarthritis (OA) is the most common disabling joint condition affecting elderly adults and it also has a significant impact on adults of working age. The aetiology remains unclear: genetic factors account for approximately half the variation in expression of OA and several predisposing genetic variants have been discovered [1]. Of the environmental risk factors that have been identified, an influence of body mass index (BMI) is now well established [2], particularly at the knee. The precise mechanism of action of BMI - via mechanical factors such as malalignment [3,4] or hormonally through leptin [5] or adiponectin [6] - and of other factors in the environment remains uncertain.

It is possible that the association between BMI and OA reflects risk factors that are inherent in diet. A role for micronutrients in both the incidence and progression of OA, particularly at the cartilage level, has been postulated for some time. Oxidants have been shown to be involved in the cartilage damage [7] and the reactive oxygen species scavenger superoxide dismutase is reduced in both human and animal models of OA [8]. Reactive oxygen species have been shown to influence both normal chondrocyte activity and cartilage damage [9] so an important role for antioxidants is widely postulated [10]. The effect of individual dietary micronutrients *in vivo*, however, remains uncertain and there are several potential explanations for this. Estimation of vitamin intake in the diet is technically difficult and the sample sizes of many studies to date have been small. The Framingham group has examined a large sample for micronutrient intake associated with both incident and progressive knee OA. Results suggested a protective effect of vitamin D on progression of knee OA [11] as well as protective effects of vitamin C, beta carotene and vitamin E [12].

The study of dietary data presents several difficulties. These include separating individual components from other, correlated foods in the diet and separating dietary factors from other lifestyle factors. To address these issues in the present study, we have examined overall patterns of dietary intake rather than individual dietary components. This method of using food patterns provides a more realistic overall measure of exposure to groups of correlated nutritional components. The analysis of data from twins, through their inherent matching of age, lifestyle and genetic background, allows the direct influence of nutritional components to be distinguished from associated lifestyle factors in the shared environment.

In this study we determined whether particular patterns of food intake are associated with prevalent OA at the hand, hip, and knee in a large volunteer cohort of healthy female twins using the food frequency questionnaire (FFQ). This is a well validated method for determining intake of macro- and micronutrients [13]. OA is known to vary by age, sex and skeletal site so only female subjects were included in this study and OA sites were analysed separately rather than combined into a single 'OA' phenotype. The aim of this cross-sectional study was threefold. First, to identify patterns of dietary intake associated with OA at any site; second to identify individual foods associated with increased or decreased risk of OA; and third to explore, using a chondrocyte-like cell line, putative mechanisms of action of compounds contained in the individual foods identified.

## Methods

### The twin sample

The epidemiology part of the study was carried out in a cohort of twins that has been recruited over the last 16 years by national media campaigns. Registered twins are sent regular questionnaires concerning a wide range of health and lifestyle traits. Twins are also invited to attend St Thomas' Hospital, King's College London http://www.twinsuk.ac.uk for imaging and other studies, and to donate biological material including blood [14]. Where possible, twins are not made aware of the precise hypothesis being tested prior to recruitment to a study. Participants are not selected, for example, for back or knee pain. Twins having previous joint replacement were excluded from the study. Zygosity had been determined by questionnaire [15], but where uncertain it was confirmed by multiplex DNA fingerprint genotyping. The participants of the TwinsUK Registry have been shown not to differ from age-matched singleton women in the distribution of common traits and outcomes, including bone mineral density [16] and to have dietary intakes comparable to other Western populations [17]. All participants gave written, informed consent and St Thomas' Hospital Ethics Committee approval had been obtained.

### Dietary and other data

Twin volunteers who had previously attended for imaging were sent the 131-item FFQ by post for completion without reference to their co-twin. This was the EPIC questionnaire which has been validated against biomarkers and levels of ascorbic acid [18]. The 131 food items formed 54 food groups, defined by similar nutrient content and culinary use. Nutrient intake was calculated from an established nutrient database. FFQ responses in twins were analysed by principal components analysis as described previously [17]. This revealed that dietary patterns were captured by 5 principal components of food consumption (these were named "fruit and vegetable", "high alcohol", "traditional English", "dieting" and "low meat"). Body mass index (BMI) was calculated by dividing weight (kg) by the square of height (m). Subjects also completed lifestyle questionnaires that included questions on physical activity which was coded as inactive, moderately active and active.

### Radiological assessment

Plain radiographs were taken of the hands, hips and knees using standard techniques and were coded for joint space narrowing (JSN) and osteophytes (OP) using the method of Kellgren and Lawrence as reported previously [19].

### In vitro studies

#### Cell culture

The SW1353 human chondrosarcoma cell line was purchased from the American Type Culture Collection (no. HTB-94) and were routinely cultured in Dulbecco's modified Eagle medium (DMEM, Invitrogen) containing 10% foetal bovine serum (FBS, Invitrogen), 2 mM glutamine, 100 IU/ml penicillin and 100 μg/ml streptomycin. Serum-free conditions used identical medium without FBS.

#### Cytotoxicity and Apoptosis

Cytotoxicity and apoptotic effects of diallyl disulphide (DADS) across dose ranges used were assessed using the CytoTox 96^® ^Non-Radioactive Cytotoxicity Assay and Caspase-Glo^® ^3/7 Assay kits as described by the manufacturer's instructions (Promega Ltd, Hampshire, UK).

#### Inhibition of histone deacetylase activity

Inhibition of histone deacetylase (HDAC) activity by DADS was tested in the SW1353 cell line. Histone, alpha tubulin and global acetylation status was examined by Western blotting. Cells were plated at 2 × 10^5 ^cells per well of a 6-well plate and left to adhere overnight followed by 12 hour serum starvation. Cells were treated with DADS for 30 minutes to 6 hours at 2.5 - 10 μM. Trichostatin A (50 ng/ml) or sodium butyrate (5 mM) were added as positive controls for HDAC inhibition. Cells were washed in ice cold PBS and whole cell lysates were harvested in sample buffer (0.058 M Tris-HCl, pH 6.8; 5% v/v glycerol; 1.7% w/v SDS). Cell lysates were sonicated at 25 kHz, 100 W for 5 seconds and total protein was quantified using a BCA assay (Pierce^®^, Thermo Scientific, Northumberland, UK). Following protein quantification, 50 mM dithiothreitol reducing agent was added and samples were boiled at 90°C prior to protein separation by SDS-PAGE. Proteins were transferred to PVDF membrane and probed for total histone 3, acetylated histone 3, α-tubulin, acetylated α-tubulin, lysine and acetylated lysine (New England Biolabs Ltd, Hertfordshire, UK). All primary antibodies were used at 1/1000 as described by the manufacturer's instructions. Swine anti-rabbit HRP-conjugated secondary antibody (DAKO UK Ltd, Cambridgeshire, UK) was used to detect the proteins of interest, and visualised using LumiGLO reagent (New England Biolabs Ltd, Hertfordshire, UK) and exposure to Kodak Biomax MS film (Sigma-Aldrich, Dorset, UK).

#### Gene expression

The impact of DADS treatment on cytokine-induced metalloproteinase gene expression was assessed in the SW1353 cell line by TaqMan^® ^qRT-PCR. Cells were plated at 1 × 10^4 ^cells per well of 96-well plate, allowed to adhere overnight followed by 12 hour serum starvation. Cells were treated with 2.5-10 μM DADS in the presence or absence of cytokines IL1 (5 ng/ml) and oncostatin M (10 ng/ml) (R&D Systems Europe Ltd, Abingdon, UK). DADS was added 30 minutes prior to cytokine stimulation. The experiment was carried out in quadruplicate. Cells were washed in ice-cold PBS twice and harvested into 30 μl of Cells-to-cDNA™ II cell lysis buffer (Ambion^®^, Applied Biosystems, Warrington, UK). Endogenous RNases were inactivated and cell lysates DNase I treated according to kit instructions for Cells-to-cDNA™ II (Ambion^®^, Applied Biosystems, Warrington, UK). DNase I-treated lysates (8 μl) were transferred to a fresh 96-well PCR plate and primed for reverse transcription using 10 mM dNTP mix (2.5 mM of each) (Bioline, London, UK) and 200 ng random primers (Invitrogen Ltd, Paisley, UK) at 70°C for 5 minutes. Reverse transcription was carried out in a total volume of 20 μl using 100U M-MLV reverse transcriptase (Invitrogen Ltd, Paisley, UK) according to the manufacturer's instructions in the presence of 40U RNasin (Promega Ltd, Hampshire, UK). The total volume of cDNA was made up to 50 μl by adding 30 μl nuclease-free water.

Relative quantification of genes was performed using the ABI Prism 7700 sequence detection system (Applied Biosystems, Warrington, UK) in accordance with the manufacturer's protocol. PCR reactions used 5 μl of reverse-transcribed RNA (a 10-fold dilution of cDNA was used for *18S *analyses), 50% TaqMan 2X Master Mix (Applied Biosystems, Warrington, UK), 100 nM of each primer and 200 nM of probe in a total volume of 25 μl. Conditions for the PCR reaction were as follows: 2 minutes at 50°C, 10 minutes at 95°C, 40 cycles of 15 seconds at 95°C, and 1 minute at 60°C. Sequences for *MMP *primers and probes are as described [20]. Relative quantification is expressed as 2^-ΔC^T, where ΔC_T _is C_T_(target gene) - C_T_(18S). The threshold cycle (C_T_), the cycle number at which signal is detectable above the baseline.

### Analytical approach

The pattern scores used as the main dietary variables were based on food intakes for which the residual method had been used to make the consumption of each food or nutrient independent of total energy intake [21]. Where individual foods were used, these were also residual-adjusted for total energy. Kellgren-Lawrence scores were coded as 0 and 1 versus 2 or higher (2+). All models were adjusted for age, BMI, smoking and physical activity. The standard errors derived from all models were adjusted for clustering by twin pair.

Logistic regression modelling was used to examine the association between Kellgren and Lawrence score and food pattern score: analyses were first undertaken treating the twins as individuals, allowing comparison with results from singleton populations. In the tables below, model 1 shows the odds ratio for OA from logistic regression models for a one standard deviation (SD) increase in the dietary intake.

Secondly, following the approach of Begg and Parides [22], the effect on OA of each dietary factor was examined in a model that included the twin pair mean for the dietary variable, in addition to the individual twins' values. In the logistic regression models, results from model 2 can be interpreted as the odds ratio for OA corresponding to a 1 SD increase in the individual's dietary factor, holding fixed the pair average. A key point is that these models enable us to examine confounding by family (pair) level influences. The pair mean of the dietary factor acts as a surrogate for influential family-level attributes, both environmental and genetic. Thus, if an association remains after adjustment for the pair mean values of a dietary variable, a robust relationship is suggested which is not due to shared genotype or environmental confounding.

## Results

Data were available for between 654 and 1082 individual twins depending on anatomical site studied (Table 1): for reasons of funding, twin numbers vary for the plain films of the hands, hips and knees. Twins were female with mean age of 58.9 (range 45.7-77.0) yrs overall. Mean BMI was 25.6 kg/m^2 ^(standard deviation = 4.0 kg/m^2^), showing that the twins were not, on the whole, obese. Considering the OA phenotypes, 14.1%, 27.8% and 58% of twins were considered 'affected' at the three sites hip, knee and hand respectively (Table 1). MZ twins comprised 41% of the sample. Dietary intake was based on multiple food questionnaires completed at different time points for the majority of these twins (68%). The mean interval between the first and last food questionnaires by those who completed more than one was 9.1 years.

**Table 1:** Characteristics of the twins

	n	Mean	SD
Age (y)	1096	58.9	5.8
BMI (kg/m^2^)	1096	25.6	4.0
			
Hip Kellgren-Lawrence score	654	0.63	1.5
Knee Kellgren-Lawrence score	912	1.00	1.7
Hand Kellgren-Lawrence score	1082	5.63	7.6

		n	%

Hip Kellgren-Lawrence score	0,1	562	85.9
	2+	92	14.1
			
Knee Kellgren-Lawrence score	0,1	671	72.2
	2+	259	27.8
			
Hand Kellgren-Lawrence score	0,1	456	42.0
	2+	630	58.0
			
Zygosity	Monozygous	454	41.4
	Dizygous	642	58.6
			
Physical activity	Inactive	365	33.3
	Moderately active	532	48.5
	Active	199	18.2
			
Number of food frequency	1	351	32.0
questionnaires used	2	485	44.3
	3	236	21.5
	4	24	2.2

BMI represents body mass index, n sample size, SD standard deviation

Table 2 shows the associations of each OA site with the five patterns of dietary exposure. Odds ratios for a Kellgren-Lawrence score of 2 or more are expressed for two models: model 1 treats the twins as individuals; model 2 includes both the individual observations (OR shown) and the twin pair mean of the dietary variable (OR not shown). This allows some examination of possible confounding by environmental or genetic factors shared by twins. Analysis of dietary patterns shows the fruit and vegetable pattern to have a significant protective effect (p = 0.022) for hip OA in model 1, even after adjustment for age, BMI and physical activity (Table 2). An association with the individual intakes is shown, and remained even after adjustment for twin-pair intake means (model 2). No association was seen with the twin pair mean itself in model 2 (results not shown) suggesting that the relationship is a real one and not due to confounding by shared factors.

**Table 2:** Logistic regression analyses of pattern scores and Kellgren-Lawrence scores

*Site*	*No. twin pairs*	*Dietary pattern*	*Model 1*	*Model 2*
			*OR (95% CI)*	*OR (95% CI)*
Hip	327	Fruit and vegetable pattern score	***0.724 (0.549,0.955)***	***0.609 (0.388,0.958)***
		High alcohol pattern score	1.073 (0.856,1.345)	1.048 (0.707,1.552)
		Traditional English pattern score	1.072 (0.868,1.323)	1.093 (0.806,1.483)
		Dieting pattern score	1.008 (0.793,1.282)	0.877 (0.619,1.242)
		Low meat pattern score	1.099 (0.881,1.371)	1.433 (0.995,2.065)
Knee	456	Fruit and vegetable pattern score	1.107 (0.939,1.306)	1.028 (0.781,1.353)
		High alcohol pattern score	1.039 (0.880,1.226)	1.029 (0.788,1.346)
		Traditional English pattern score	1.127 (0.964,1.318)	1.245 (0.987,1.571)
		Dieting pattern score	1.088 (0.918,1.290)	1.158 (0.891,1.506)
		Low meat pattern score	0.973 (0.833,1.136)	1.042 (0.817,1.330)
Hand	541	Fruit and vegetable pattern score	0.983 (0.861,1.122)	1.009 (0.844,1.206)
		High alcohol pattern score	1.028 (0.901,1.174)	1.116 (0.915,1.362)
		Traditional English pattern score	1.033 (0.906,1.179)	0.988 (0.831,1.176)
		Dieting pattern score	1.015 (0.894,1.153)	0.975 (0.827,1.149)
		Low meat pattern score	0.940 (0.825,1.069)	0.857 (0.723,1.017)

Model 1: individual pattern scores only; model 2: individual pattern scores and twin pair means in the same model

All models adjusted for age, BMI and physical activity; Kellgren-Lawrence scores coded as 0 and 1 versus 2+

This was an exploratory analysis and, although the use of pattern scores rather than individual food items meant that comparatively few hypotheses were investigated, we were aware of conducting multiple comparisons. We therefore used simulations, based on swapping OA scores randomly between twin pairs (thus keeping the twin-based structure of both the food and OA data intact), and carrying out the same analyses for 10,000 replications. No results were significant after this permutation-based adjustment for multiple comparisons (results not shown).

Once the individual dietary patterns have been shown to be potentially associated with OA, further investigation was made of the individual dietary components contributing to the pattern. A high value for the fruit and vegetable dietary score indicates frequent intakes of fruit, alliums (members of the garlic family) and cruciferous vegetables, with low intakes of fried potatoes. Of those that contributed to the association between the fruit and vegetable pattern and hip OA, consumption of non-citrus fruit (p = 0.015) and alliums (garlic, leeks and onions) (p = 0.029) showed the strongest protective association with hip OA (Table 3).

**Table 3:** Logistic regression analyses of foods with large coefficients in the fruit and vegetable pattern score for hip

*Food item*	*Model 1*	*Model 2*
	*OR (95% CI)*	*OR (95% CI)*
Allium vegetables^1^	***0.748 (0.577,0.971)***	***0.702 (0.506,0.973)***
Cruciferous vegetables^2^	0.819 (0.640,1.049)	0.719 (0.466,1.110)
Green leafy vegetables^3^	1.024 (0.810,1.296)	1.051 (0.725,1.525)
Yellow vegetables^4^	0.918 (0.732,1.150)	1.117 (0.751,1.661)
Other vegetables^5^	0.962 (0.736,1.257)	0.907 (0.614,1.339)
Citrus fruit^6^	0.858 (0.570,1.293)	0.784 (0.565,1.089)
Non-citrus fruit^7^	***0.676 (0.493,0.927)***	***0.560 (0.396,0.790)***
Chips and roast potatoes	1.173 (0.959,1.434)	1.314 (0.940,1.836)

^1 ^garlic, onions and leeks ^2 ^cabbage, broccoli, Brussels sprouts, cauliflower and coleslaw ^3 ^green salad and spinach ^4 ^carrots and tomatoes ^5 ^parsnips, marrow, watercress, sweetcorn, avocado, beetroot, mushrooms and sweet peppers ^6 ^oranges and grapefruit ^7 ^apples, bananas, pears, peaches, melon, grapes, tinned fruit and dried fruit

Model 1: individual food intakes only; model 2: OR shown for individual food intakes, but obtained with twin pair mean intakes in the same model

All models also adjusted for age, BMI and physical activity; Kellgren-Lawrence scores coded as 0 and 1 versus 2+

All foods listed had high positive loadings in the fruit and vegetable pattern, except chips and roast potatoes, which had a high negative loading

**Figure 1:**
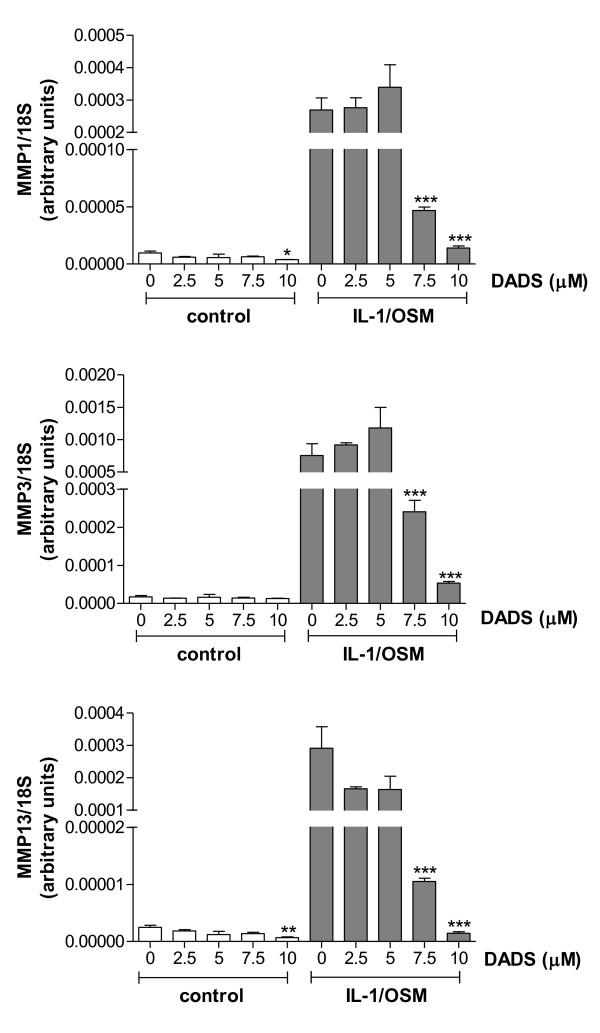
**Diallyl disulphide regulates the expression of key MMP genes**. SW1353 chondrosarcoma cells were treated +/- interleukin-1 (5 ng/ml) and oncostatin M (10 ng/ml), IL-1/OSM, in the absence or presence of increasing doses of diallyl disulphide (DADS) for 6 hrs. Gene expression was measured using qRT-PCR and normalized to the 18S housekeeping gene. Data is shown as mean +/- SEM (n = 3). Statistics are from t-test: *, p < 0.05; **, p < 0.01; ***, p < 0.001.

Garlic and other allium vegetables contain a number of bioactive compounds (reviewed in [23]). When garlic is crushed, damaged or chopped, an enzyme alliinase is activated and acts upon alliin to form allicin. Allicin is unstable and further decomposes to yield sulphides, ajoene and dithiins. Several of these compounds have shown biological activities with diallyl sulphides most extensively investigated - particularly diallyl disulphide (DADS) [24]. Interleukin-1 (IL-1), and particularly the combination of IL-1 and oncostatin M (OSM), has been shown to cause the destruction of articular cartilage both *in vitro *and *in vivo *by inducing of expression and activation of matrix-degrading metalloproteinase enzymes, eg the matrix metalloproteinases (MMPs) [25,26]. Broad spectrum histone deacetylase (HDAC) inhibitors have been shown to repress this MMP induction and are chondroprotective *in vitro *and *in vivo *[27]. DADS is reported to have HDAC inhibitor activity [28] so we measured both the activity of DADS to induce acetylation of histones, and also its ability to repress the IL-1 or IL-1/OSM-induced expression of key MMPs, MMP-1 and -13 (collagen-degrading enzymes) and MMP-3 (an activator of proMMPs) in a model cell line. Figure 1 shows that DADS dose-dependently represses the IL-1/OSM-induced expression of all three MMPs. Under these conditions DADS shows no toxicity (data not shown), nor does it alter acetylation as determined by Western blot (data not shown).

## Discussion

The investigation of diet in OA is an area fraught with methodological issues and there are few large-scale studies in the literature. This study is among the first and is unique in its use of dietary patterns and population-based twins to overcome some of the major technical difficulties of diet epidemiology in complex traits. The chief finding is that a 'healthy diet' containing high intake of fruit and vegetables (and alliums in particular - the onion genus including garlic, onions, shallots, chives and leeks) are protective for hip OA. The p-values of individual nutrients are not small and do not survive the more stringent methods of correction for multiple testing. However, the overall dietary patterns are consistent with the results from the study of individual nutrients which show fruit and vegetables to be inversely associated with hip OA.

The main limitation of this study is its cross sectional design. This has been addressed, to some extent, by the use of FFQ at multiple time points for the majority of twins, a technique known to increase the signal to noise ratio. Although we have not performed a longitudinal study in the true sense of the design, readings over several time points were averaged, reducing the noise in measured variables and improving their accuracy. Determining the direction of cause and effect is clearly difficult without longitudinal or intervention studies so we cannot be certain of the direction of effect: twins in pain from OA, for example, may modify their dietary content or reduce their caloric consumption. However, given the chronicity of OA and the early stages of OA detected in this healthy volunteer cohort, it would seem more likely that the diet precedes the radiographic changes. As with all studies of diet there are inherent flaws and limitations in the use of FFQs for measuring dietary intake: instruments such as diet diaries or dietary recalls are considered more accurate [29]. The FFQ can, however, be used reliably to rank individuals by intake, and is more representative of diet over extended periods. Dietary patterns obtained from FFQs have been shown to correlate well with patterns derived from dietary records [30,31] and can measure food intake for a period of about a year, but clearly do not reflect lifetime dietary habits. One of the strengths of studying twins is their intrinsic matching; differential recall is likely to be similar in twins, and our model can examine the influence of pair-level, confounding lifestyle factors. The associations seen were independent of BMI. These twin volunteers have been shown to be similar to a singleton volunteer cohort for a number of different disease and lifestyle traits [16]: furthermore, their dietary behaviour has also been shown to be representative of the general population [17].

Because of the difficulties of this type of epidemiological study, we sought to validate the findings by investigating individual bioactive compounds. One of the active compounds found in allium vegetables, diallyl disulphide is from the thiosulphonate family whose members are reported to act as inhibitors of histone deacetylases (HDAC) [32]. HDACs are believed to modify gene expression by influencing acetylation/deacetylation of histones and other proteins. Broad spectrum HDAC inhibitors have also been shown to block the expression and activity of key matrix-degrading proteases [27]. Hence, we conducted proof of concept laboratory studies using DADS in a chondrosarcoma cell line, measuring both cellular acetylation and its ability to repress the IL-1/OSM-induced expression of key matrix-degrading metalloproteinases as a surrogate for the destruction/protection of articular cartilage. We showed that DADS had no effect on cellular acetylation in these cells (data not shown). However, DADS showed a dose-dependent repression of induced MMP expression. A related compound diallyl sulphide (DAS), also from allium vegetables, has previously been shown to inhibit IL-1 or urate crystal-induced cyclooxygenase 2 expression in both chondrocytes and synovial cells *in vitro *and in a rat model of joint inflammation [33]. We have conducted preliminary experiments with this compound which showed that it can also dose-dependently repress induced MMP expression in a similar manner to DADS (data not shown). These findings show the potential mechanistic links between the consumption of allium vegetables and joint metabolism and inflammation, albeit independent of histone acetylation. The molecular mechanisms by which these allyl sulphides mediate repression of metalloproteinase expression remain unknown, but may involve their ability to act as antioxidants, inhibition of NFκB or inhibition of MAP kinase activity.

As the subjects studied here are healthy volunteers, our study represents an examination of early radiographic OA - in many cases pre-symptomatic. It may be for this reason we found a relationship with prevalent disease when no such relation was reported by the Framingham group. Their study was of similar sample size and used questionnaire information to ascertain vitamin D intake (as well as serum levels), and showed OA progression was threefold higher in the middle and lower tertile of vitamin D intake [12]. There was no consistent association with incident disease and only the knee joint was examined. The present study has the advantage of using twin pairs, detail on a greater number of food groups as well as OA at a number of anatomical sites. It is surprising that a dietary link was identified with degenerative change at the hip but not at the hand or knee. This may be a true finding: it is well recognised that both genetic and environmental risk factors for OA differ across body sites, and this observation may reflect local differences in biomechanical or environmental influence on the disease. Alternatively this may be a reflection of differential sensitivities of radiographs at these sites and differences in statistical power.

A particular strength of this study lies in the analysis of dietary patterns, rather than individual foods, in the first instance. With the analysis of a small number of pattern scores that summarise consumption of a large number of correlated foods, there is less of an issue with multiple testing than if hundreds of correlated intakes had been tested individually in a hypothesis-free study. In addition, the twin design offers benefits over other studies particularly in the study of environmental effects because the pairing of twins controls completely for age and sex and, to some extent, for hormonal influences as well as many measured and unmeasured confounders.

## Conclusions

Early disease intervention or even primary prevention of OA represents an important goal in the management of this common disease. Results presented here raise the possibility that progression of hip OA might be amenable to dietary modification or nutriceutical intervention. Our findings throw the spotlight onto the allium family and potential disease modification via bioactive compounds from this plant family. If these results are confirmed by independent replication, then a dietary intervention trial would be a reasonable next step. OA is clearly related to BMI, which is projected to increase rapidly in prevalence. These data shed intriguing light on dietary influences and may be of importance in formulating accurate public health messages in the future.

## Competing interests

The authors declare that they have no competing interests.

## Authors' contributions

All authors have read and approved the final manuscript. FW was involved in interpretation of the data, drafting and revising manuscript and has given final approval. JS analysed and interpreted the data, revised manuscript and gave final approval. TS was involved in data collection, revision of manuscript and gave final approval. AE was involved in design, data collection, revision of manuscript and gave final approval. IMC was involved in the design of the laboratory studies, interpretation of data, revision of manuscript and gave final approval. AM was involved in study design, collection of data, analysis and interpretation, drafting and revision of manuscript and gave final approval.

## Pre-publication history

The pre-publication history for this paper can be accessed here:

http://www.biomedcentral.com/1471-2474/11/280/prepub
